# Relative importance of psychosocial and organizational predictors of burnout among European researchers: a dominance analysis

**DOI:** 10.3389/fpubh.2026.1900453

**Published:** 2026-10-19

**Authors:** May Thet Nu Noe, Pascal Wild, Stefan T. Mol, Irina Guseva Canu

**Affiliations:** 1 Centre for Primary Care and Public Health (Unisanté), University of Lausanne, Lausanne, Switzerland; 2 Amsterdam Business School, University of Amsterdam, Amsterdam, Netherlands

**Keywords:** higher education, multiple imputation, occupational health, organizational determinants, relative importance analysis, researchers

## Abstract

**Objectives:**

Burnout is increasingly reported among researchers in European academia; however, the relative importance of its psychosocial and organizational determinants across career stages remains insufficiently understood. This study aimed to rank the relative importance of a defined set of predictors of burnout among junior and senior researchers.

**Methods:**

Cross-sectional data were drawn from the European Researcher Mental Health Observatory (ReMO) Staircase survey (*N* = 4,026). Burnout was assessed using the 12-item Burnout Assessment Tool. Analyses were conducted separately for junior and senior researchers. Missing data were addressed using multiple imputation by chained equations. Dominance analysis estimated the relative importance of twelve predictors among junior researchers and ten among senior researchers based on their contribution to explained variance (R^2^), with bootstrapped confidence intervals computed for all predictors.

**Results:**

The models explained substantial variance in burnout (*R^2^* = 0.485 for junior researchers; *R^2^* = 0.515 for senior researchers). Resilience and role clarity emerged as the most important predictors in both groups, followed by work–family conflict and work-related stress. Illegitimate tasks showed somewhat greater dominance among senior researchers (in a comparison restricted to the ten predictors shared by both groups), while other predictors contributed similarly across career stages. Confidence intervals indicated that several mid-ranked predictors were not clearly separable from one another. The overall pattern of predictor dominance was broadly consistent across career stages and analytical approaches.

**Conclusion:**

Burnout in academia reflects systemic features of modern higher education, including high workload, role ambiguity, administrative burden, and competitive evaluation systems. These results support a shift from individual-focused interventions toward structural reforms. A Total Worker Health approach is recommended, emphasizing improved role clarity, workload regulation, job stability, and more balanced reward systems to promote sustainable academic careers.

## 1 Introduction

Higher education institutions in Europe are increasingly positioned as key actors in the global knowledge economy, contributing to innovation, economic development, and international competitiveness (1, 2). In recent decades, universities have undergone a substantial structural transformation driven by globalization, technological advancement, and expanding societal expectations. These shifts have been accompanied by the rise of internationalization agendas and accountability frameworks that have reshaped institutional governance and priorities (2). In this evolving context, institutional sustainability depends not only on performance outcomes but also on the well-being of the academic workforce (3, 4).

The nature of academic work has changed considerably alongside these transformations. Roles that were once characterized by autonomy and relatively stable career trajectories now involve increasingly complex and diversified responsibilities, including research, teaching, administration, supervision, and institutional service (5). At the same time, growing administrative and managerial requirements, such as reporting obligations, and quality assurance processes, have increased the administrative workload (6). Together, these developments have intensified work demands, fragmented tasks, and reduced clarity in role expectations, making it more difficult for academics to balance competing responsibilities.

These changes have contributed to rising levels of occupational strain and burnout in academia. Estimates of burnout among academic staff vary considerably depending on the population studied, the measurement instrument, and the cut-off applied. For example, Mohamed et al. (7) assessed burnout among Malaysian academics using the Copenhagen Burnout Inventory, while Guthrie et al. (3) highlighted substantial heterogeneity in burnout estimates across research environments. Workload, administrative burden, and work–family conflict consistently emerge as central predictors of burnout among academic staff, with evidence across European and international contexts highlighting work–family conflict as particularly impactful (8, 9). Furthermore, broader comparative scoping data across professional and educational populations indicate that organizational and psychosocial stressors function as critical determinants of occupational burnout (10). Beyond workload, psychosocial conditions, including limited institutional support, workplace conflicts, and low role clarity, further exacerbate risk, whereas supportive collegial environments and participative leadership appear to protect against it (8, 9). In addition, the growing digitalization of academic work has fostered an “always-on” culture, blurring boundaries between professional and personal life (11). At the structural level, competition for research funding is intense: in the European Research Council’s 2024 Synergy Grant call, 57 of 548 proposals were funded, a success rate of approximately 10% (12). Temporary contracts are also prevalent, particularly among early-career researchers, and further contribute to burnout (13).

Burnout has significant consequences for both individuals and institutions, being associated with reduced performance, turnover intentions, and adverse mental health outcomes among academics (14, 15). At the system level, evidence from Europe shows that education professionals report high levels of stress, fatigue, and mental health problems, highlighting the need for policy attention to mitigating psychosocial risks in higher education (15, 16).

While a growing body of research has identified multiple psychosocial and organizational predictors of burnout in academia, including workload, role clarity, job insecurity, interpersonal conflict, organizational support, job control, and sense of community (3, 17), these factors are often examined in isolation. This piecemeal approach limits understanding of their relative importance and, consequently, the prioritization of intervention opportunities. The Job Demands–Resources model proposes that burnout develops primarily through a health impairment process driven by job demands (18, 19), and meta-analytic evidence indicates that hindrance demands, such as role ambiguity, are more strongly associated with burnout than job resources (20, 21). We therefore expected demand-related predictors, in particular low role clarity, work–family conflict, and work-related stress, to rank above job resources such as job control and sense of community. The present study applies dominance analysis (22, 23) to rank the relative contribution of twelve psychosocial and organizational predictors to explained variance in burnout among junior researchers and ten predictors among senior researchers. Because dominance rankings are conditional on the predictors included, the resulting hierarchy applies to the retained predictor set rather than to all possible determinants of burnout.

## 2 Methods

### 2.1 Study population and design

This cross-sectional study was conducted as part of the Researcher Mental Health Observatory (ReMO; COST Action CA19117). The target population comprised researchers working in academic settings across different career stages, including R1 (PhD students/candidates), R2 (postdoctoral researchers and junior lecturers), R3 (senior lecturers and assistant/associate professors), and R4 (full professors), as defined by the EURAXESS taxonomy.

Eligible participants were individuals aged 18 years or older who were currently engaged in research activities. Individuals not working in research roles (e.g., students, administrative, or technical staff) were excluded. The flowchart of participants from survey access to the analytic samples is shown in Figure 1. The study is reported in accordance with the STROBE statement for cross-sectional studies (24); the completed checklist is provided in Supplementary File S1.

**Figure 1:**
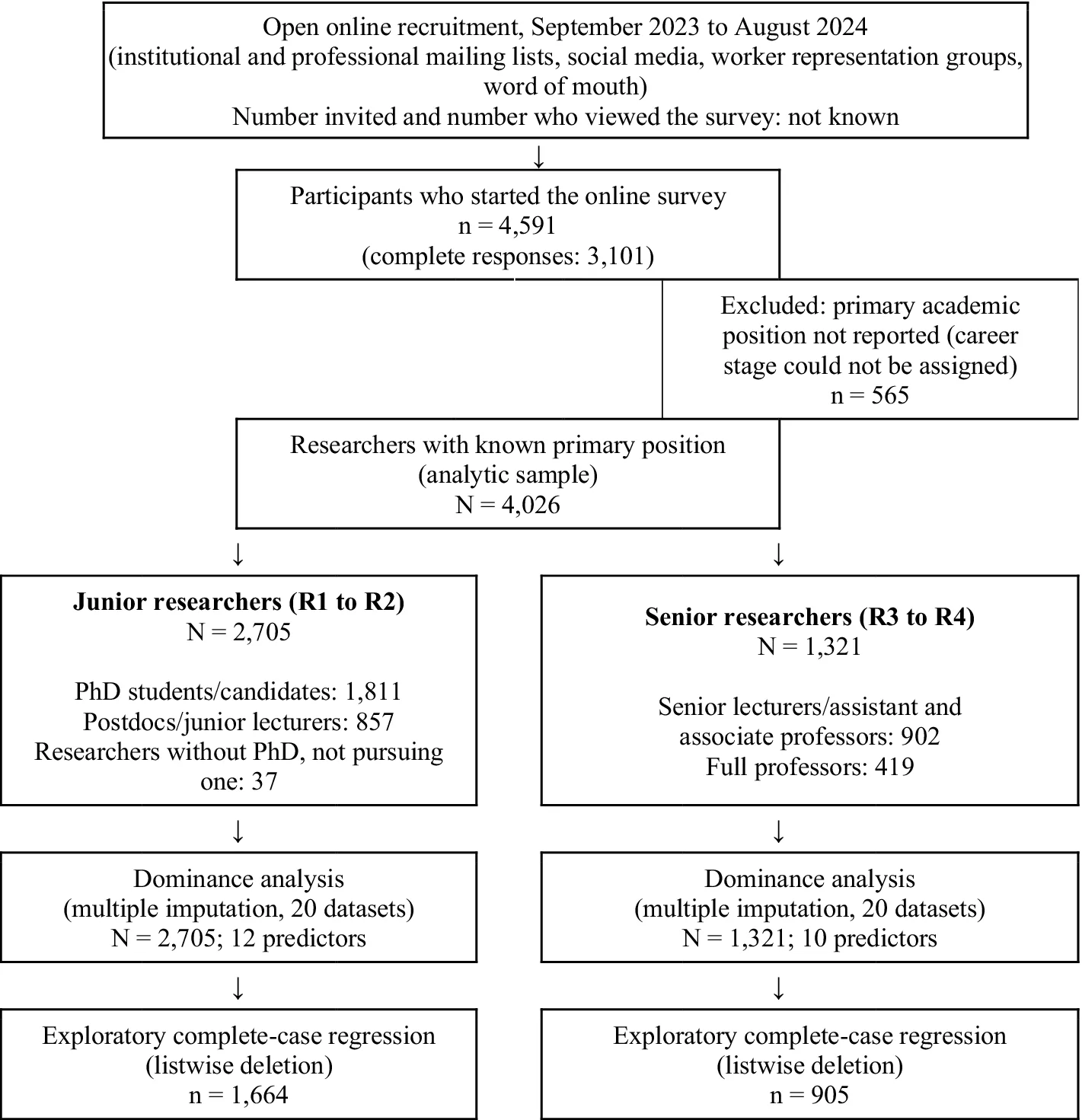
Flowchart of participants from survey responses to the analytic samples. R1 to R4 refer to the EURAXESS researcher career stages.

#### 2.1.1 Ethical approval

The study protocol was approved by the Ethics Commission of Leibniz University Hannover (EV LUH 04/2023), and all participants provided informed consent prior to participation.

### 2.2 Data collection

The survey design and data collection procedures have been described previously (25, 26). Briefly, data were collected through the STAIRCASE (Sustainable Working Conditions in Academia) survey administered via LimeSurvey from September 15, 2023 until August 26, 2024. The survey comprised five main sections: (i) demographics, (ii) mental health outcomes, (iii) working and studying conditions, (iv) the working environment, and (v) leadership and supervision. Participants were invited via institutional and professional mailing lists (61.2%), social media, worker representation groups, and word of mouth.

### 2.3 Study outcome

Burnout was the main outcome considered. It was assessed using the short 12-item version of Burnout Assessment Tool (BAT-12) (27, 28), which consists of four dimensions: exhaustion, cognitive impairment, emotional impairment, and mental distance. Items were scored on a five-point scale ranging from 1 (“never”) to 5 (“always”) and averaged to produce a mean burnout score, with higher scores indicating greater burnout. The BAT-12 has demonstrated excellent psychometric properties in previous validation studies, including strong factorial validity and reliability across occupations (29). Burnout was treated as a continuous variable in all analyses. To address potential resolution trade-offs of using a single aggregated score, dimension-level dominance checks were additionally evaluated across the four subdimensions.

### 2.4 Predictor variables

Sixteen psychosocial and organizational factors were initially considered as potential predictors of burnout, including role clarity, resilience, predictability at task, illegitimate tasks, work-related stress, work–family conflict, job insecurity, job control, interpersonal conflict, policy perception, sense of community, leader’s lack of care, leader’s excessive demands for results, emotional support, instrumental support, and supervisor integrity. Variables for which more than 30% of responses were missing at the overall scale (factor score) level were excluded from the analysis. This decision follows established recommendations for handling missing data in survey research, which suggest that variables with high levels of missingness should be removed to ensure data quality and reduce bias (30). This specific 30% cut-off is a pragmatic threshold commonly applied in practice rather than a value derived from that literature, and we treat it as a heuristic rather than a definitive rule. On this criterion, policy perception (46.0% missing among junior and 37.1% among senior researchers), emotional support (44.5 and 44.4%), instrumental support (45.1 and 44.7%), and supervisor integrity (35.8 and 74.7%) were excluded (Supplementary Table S1). Consequently, twelve predictors were retained for junior researchers and ten for senior researchers, for whom the two leader scales were not administered.

Working conditions differ substantially between career stages: in our sample, half of junior researchers held contracts of less than two years, whereas most senior researchers held permanent positions (Table 1), and senior researchers more often hold supervisory and leadership roles. Analyses were therefore conducted separately for junior and senior researchers. In a companion analysis of the same data, a multigroup structural equation model of the Job Demands-Resources framework, including the BAT-12 dimensions, did not achieve metric or scalar measurement invariance across junior and senior researchers (31). The present analyses therefore compare the rank order of predictors within each group rather than parameters or means across groups.

**Table 1:** Demographic and professional characteristics of junior and senior researchers.

Characteristics	Junior researchers (*N* = 2,705)	Senior researchers (*N* = 1,321)
Age		
<28	612 (22.6%)	6 (0.5%)
28–33	1,096 (40.5%)	29 (2.2%)
34–39	555 (20.5%)	182 (13.8%)
40–45	259 (9.6%)	341 (25.8%)
46–51	107 (4.0%)	297 (22.5%)
52–57	42 (1.6%)	222 (16.8%)
>57	23 (0.9%)	239 (18.1%)
Missing	11 (0.4%)	5 (0.4%)
Gender		
Female	1,729 (63.9%)	787 (59.6%)
Male	926 (34.2%)	514 (39.0%)
Not reported	50 (1.8%)	20 (1.5%)
Contract termination		
Within 12 months	855 (31.6%)	74 (5.6%)
1–2 years	517 (19.1%)	42 (3.2%)
2–3 years	420 (15.5%)	59 (4.5%)
>3 years	146 (5.4%)	60 (4.5%)
Permanent	305 (11.3%)	809 (61.2%)
No contract/stipend or not reported†	462 (17.1%)	277 (21.0%)

† Includes respondents who reported having no contract or stipend and those who did not answer the question.

All predictors were computed as factor scores. Items with reverse wording were recoded before scoring, so that higher scores indicate more of the construct named in Table 2. Two scales are named according to their item content: the job insecurity scale was keyed towards job security, and the leader lack of care items were positively worded; these variables are therefore labelled job security and leader’s care, with higher scores indicating greater security and greater care. Instrument sources, scoring direction, and missing data are summarized in Table 2.

**Table 2:** Overview of study variables and missing data across junior and senior researchers.

Predictor	Measure (source)	Items	Higher score indicates	Junior missing, *n* (%) *N* = 2,705	Senior missing, *n* (%) *N* = 1,321
Role clarity	COPSOQ I and III * (52, 53)	4	Greater role clarity	532 (19.7)	306 (23.2)
Resilience	Brief Resilience Scale (54)	6	Greater resilience†	265 (9.8)	158 (12.0)
Predictability	COPSOQ III (53)	3	Greater predictability†	526 (19.4)	284 (21.5)
Illegitimate tasks	Bern Illegitimate Tasks Scale (55)	4	More frequent illegitimate tasks	542 (20.0)	299 (22.6)
Work-related stress	HSE Management Standards Indicator Tool (56)	3	Greater work-related stress	518 (19.1)	278 (21.0)
Work–family conflict	Work–Family Conflict Scale (57)	5	Greater work–family conflict	561 (20.7)	294 (22.3)
Job security	Job Insecurity Scale (58)	4	Greater job security†	612 (22.6)	298 (22.6)
Job control	Job Diagnostic Survey (59)	3	Greater job control	499 (18.4)	280 (21.2)
Interpersonal conflict	KIWEST (60)	3	Greater interpersonal conflict	569 (21.0)	302 (22.9)
Sense of community	COPSOQ III (53)	3	Stronger sense of community	515 (19.0)	276 (20.9)
Leader’s excessive pressure for results	Harmful Leader Behaviour Scale (61)	6	Greater pressure for results	773 (28.6)	Not administered
Leader’s care	Harmful Leader Behaviour Scale (61)	4	Greater leader care	693 (25.6)	Not administered
Burnout (outcome)	BAT-12 (27, 28)	12	Greater burnout	212 (7.8)	122 (9.2)

All missing values were imputed in each of the 20 imputed datasets, so the number imputed equals the number missing. All variables except the two leader scales were used for both junior and senior researchers; the leader scales refer to supervisory leadership and were not administered to senior researchers, who typically occupy leadership positions. COPSOQ, Copenhagen Psychosocial Questionnaire; HSE, Health and Safety Executive; KIWEST, Knowledge-Intensive Work Environment Survey Target. *One item appears only in COPSOQ I; the other three are included in both COPSOQ I and COPSOQ III.

Three of the four excluded measures (supervisor integrity, emotional support, and instrumental support) are recognized protective resources within the job demands-resources framework (18, 19). Their exclusion reflects data completeness rather than evidence that they are unimportant, and the rankings reported below should therefore be interpreted as applying to the retained predictor set rather than as a complete inventory of burnout determinants; this is discussed further as a limitation.

### 2.5 Data management and statistical analysis

#### 2.5.1 Sample stratification and exploratory analyses

The analytical sample was stratified into junior (R1 to R2, including 37 researchers without a PhD who were not pursuing one) and senior (R3 to R4) researchers. As an initial step, multivariable linear regressions were conducted separately for each group using complete cases (*N* = 1,664 juniors; *N* = 905 seniors) to examine baseline associations between psychosocial and organizational factors and burnout. These analyses, based on listwise deletion, were intended as exploratory robustness checks rather than the primary inference corresponding to the multiple-imputation-based dominance analyses.

#### 2.5.2 Missing data handling

Because listwise deletion in the multivariable models would have removed approximately 38% of junior and 31% of senior researchers, multiple imputation was used in the main analyses to preserve statistical power and reduce bias from missing data. Missing values were handled using multiple imputation by chained equations (MICE), with 20 imputed datasets created per group (32). The imputation model included the twelve predictor factor scores for junior researchers or the ten predictor factor scores for senior researchers, together with the burnout outcome. All missing scores were imputed as continuous variables in each dataset (Table 2).

To evaluate the plausibility of the missing-at-random (MAR) assumption, a diagnostic logistic regression was estimated on the unimputed data, modelling missingness in burnout (0 = observed, 1 = missing) as a function of age category, gender, and primary academic position (Supplementary Table S2). This model included 3,942 of the 4,026 respondents; 84 were excluded because of missing age category (*n* = 16) or gender (*n* = 70). As is inherent to MAR, this diagnostic cannot rule out a relationship between missingness and the unobserved burnout values themselves. Socio-demographic covariates were not included as predictors in the dominance analysis because they were not part of the prespecified substantive predictor set; including them would also have substantially increased the number of subset models required (from 4,095 models for 12 predictors to 32,767 for 15).

#### 2.5.3 Dominance analysis strategy

As an initial exploratory step, dominance analysis was conducted using listwise-deleted data to obtain a preliminary ranking of predictors. This step was purely descriptive and informed subsequent modeling decisions.

The main dominance analyses were conducted using the domin package, version 3.6.0 (23), in Stata. The 20 imputed datasets were stacked into a single dataset per group (junior researchers, 54,100 rows; senior researchers, 26,420 rows), and all subset regression models were fitted on the stacked data. Stacking yields point estimates of R^2^ that combine information across imputations, but it inflates the apparent sample size. Therefore, stacked data were used for point estimates only, and uncertainty was quantified by bootstrapping (described below). Because the domin documentation does not describe the handling of multiply imputed data, the automated output was cross-validated by manual calculation following Azen and Budescu (22). This validation covered the five highest-ranked predictors (role clarity, resilience, work-related stress, work–family conflict, and illegitimate tasks), for which all 2^5^–1 = 31 subset models were estimated separately for junior and senior researchers and compared with the automated output (Supplementary Tables S3–S5).

Analyses included all retained predictors (12 for junior and 10 for senior researchers). General, conditional, and complete dominance statistics were estimated for each group. General dominance quantified each predictor’s average contribution to explained variance across all subset models and was used to rank predictors. Conditional dominance assessed each predictor’s contribution within models of the same size, and complete dominance evaluated whether one predictor contributed more explained variance than another across all subset models (22, 23).

To quantify the precision of the general dominance statistics, nonparametric bootstrapping was performed within imputed datasets: 100 bootstrap replications were drawn in each of imputed datasets 1 to 5, giving 500 bootstrap estimates per group, and 95% intervals were taken as the 2.5th and 97.5th percentiles of these estimates. Resampling within several imputed datasets reflects both sampling variability and part of the between-imputation variability. The number of replications was limited by computing constraints in the secure remote analysis environment through which the data are accessed; percentile intervals were chosen because they require fewer replications than bias-corrected intervals. Random seeds were 12346 to 12350 for both groups.

Because the junior model contained two more predictors than the senior model, standardized dominance percentages are shares of different totals. As a sensitivity analysis, the analysis of junior researchers was therefore repeated with the same ten predictors as the model used for senior researchers (Supplementary Table S6). As a secondary analysis, general dominance analysis was repeated for each of the four BAT-12 dimensions to assess whether predictor importance differed across dimensions of burnout.

#### 2.5.4 Discriminant validity of resilience and burnout

Because resilience emerged as the top-ranked predictor and both the Brief Resilience Scale and the BAT-12 contain negative affective and exhaustion-related content, we evaluated whether resilience’s dominance could reflect overlap with the outcome rather than a distinct predictor effect. First, Pearson correlations between resilience (mean of the six BRS items) and overall burnout and each BAT-12 dimension were estimated on the unimputed data, separately for junior and senior researchers. Second, confirmatory factor analyses (CFA) compared a one-factor model, in which all BRS and BAT-12 items loaded on a single factor, with a two-factor model, in which resilience and burnout items loaded on separate but correlated factors. Models were estimated by maximum likelihood, treating the five-point items as continuous, which is considered acceptable for items with five or more response categories (33). Models were estimated in the combined sample and separately for junior and senior researchers, and compared using the chi-square difference test (Δdf = 1) and standard fit indices (RMSEA, CFI, TLI, SRMR, AIC, BIC).

All analyses were conducted in Stata 18 SE, accessed through the DZHW secure remote analysis environment.

## 3 Results

### 3.1 Study sample description

The sample included 4,026 researchers (2,705 junior; 1,321 senior). Junior researchers were generally younger, with most aged under 34 years, whereas senior researchers were predominantly aged 40 years or older. Regarding employment conditions, junior researchers were more likely to hold short-term contracts of less than two years (50.7%), whereas senior researchers predominantly held permanent positions (61.2%). Women represented 63.9% of junior and 59.6% of senior researchers (Table 1). Country- and institution-level breakdowns were not available because the ReMO STAIRCASE data-governance agreement restricts disclosure of participant country and institutional identifiers, to protect the anonymity of participating institutions. A conventional response rate could not be computed because participants were recruited through multiple parallel channels (institutional and professional mailing lists, social media, worker representation groups, and word of mouth) for which no common invitation denominator exists. Missingness in burnout was not significantly associated with age category, gender, and primary academic position (likelihood ratio (LR) χ^2^(11) = 19.18, *p* = 0.06; Supplementary Table S2), providing no evidence against the missing-at-random assumption used for imputation (Section 2.5.2).

### 3.2 Regression results

Resilience, role clarity, job security, and sense of community were associated with lower burnout in both junior and senior researchers, whereas work-related stress, work–family conflict, and illegitimate tasks were associated with higher burnout. Overall, the models explained approximately half of the variance in burnout in both groups (49% for junior researchers and 51% for senior researchers; Table 3). This complete-case analysis represents the exploratory analysis described in Section 2.5.1 and precedes the multiple-imputation-based dominance analyses reported in Tables 4, 5, which constitute the primary analyses. Correlations among all study variables are shown in Supplementary Tables S7, S8.

**Table 3:** Multiple regression models predicting burnout among junior and senior researchers.

Variable	Junior researchers (*N* = 1,664, *R*^2^ = 0.49)		Senior researchers (*N* = 905, *R*^2^ = 0.51)	
	β (95% CI)	*p*	β (95% CI)	*p*
Role clarity	−0.14 (−0.17, −0.11)	< 0.001	−0.12 (−0.16, −0.08)	< 0.001
Resilience	−0.19 (−0.22, −0.17)	< 0.001	−0.22 (−0.25, −0.18)	< 0.001
Predictability	−0.02 (−0.06, 0.02)	0.245	−0.03 (−0.08, 0.01)	0.171
Illegitimate tasks	0.06 (0.03, 0.09)	< 0.001	0.07 (0.02, 0.11)	0.004
Work-related stress	0.08 (0.05, 0.12)	< 0.001	0.11 (0.07, 0.16)	< 0.001
Work–family conflict	0.08 (0.05, 0.11)	< 0.001	0.09 (0.05, 0.13)	< 0.001
Job security	−0.08 (−0.10, −0.05)	< 0.001	−0.13 (−0.17, −0.08)	< 0.001
Job control	−0.02 (−0.04, 0.01)	0.269	−0.01 (−0.05, 0.03)	0.660
Interpersonal conflict	0.00 (−0.03, 0.03)	0.825	−0.01 (−0.05, 0.03)	0.580
Sense of community	−0.06 (−0.09, −0.03)	< 0.001	−0.08 (−0.12, −0.03)	0.001
Leader’s care	−0.03 (−0.07, 0.00)	0.062	—	—
Leader’s excessive pressure for results	0.01 (−0.02, 0.05)	0.477	—	—

Regression models are based on complete-case analyses using listwise deletion. β, standardized regression coefficient; CI, confidence interval. Higher scores indicate more of the named construct (Table 2). The two leader scales were not administered to senior researchers because they refer to supervisory leadership by others.

**Table 4:** General dominance analysis of burnout predictors among junior researchers.

Predictor	General dominance	Standardized dominance (%)	Rank	95% bootstrap interval*
Resilience	0.1215	25.1	1	0.101 to 0.139
Role clarity	0.0726	15.0	2	0.057 to 0.088
Work–family conflict	0.0444	9.2	3	0.035 to 0.055
Work-related stress	0.0406	8.4	4	0.032 to 0.050
Job security	0.0388	8.0	5	0.031 to 0.055
Predictability	0.0325	6.7	6	0.026 to 0.041
Illegitimate tasks	0.0314	6.5	7	0.024 to 0.043
Sense of community	0.0305	6.3	8	0.023 to 0.041
Leader’s care	0.0246	5.1	9	0.018 to 0.030
Leader’s excessive pressure for results	0.0209	4.3	10	0.016 to 0.029
Interpersonal conflict	0.0155	3.2	11	0.012 to 0.021
Job control	0.0116	2.4	12	0.008 to 0.018
Overall R^2^	0.485	100.0		

Point estimates from the domin package (version 3.6.0) on the 20 stacked imputed datasets (*N* = 2,705). Standardized dominance is the general dominance statistic as a percentage of the overall R^2^. Higher scores indicate more of the named construct (Table 2). *2.5th to 97.5th percentiles of 500 bootstrap estimates (100 replications in each of imputed datasets 1 to 5).

**Table 5:** General dominance analysis of burnout predictors among senior researchers.

Predictor	General dominance	Standardized dominance (%)	Rank	95% bootstrap interval*
Resilience	0.1204	23.4	1	0.096 to 0.147
Role clarity	0.0703	13.6	2	0.055 to 0.094
Work–family conflict	0.0625	12.1	3	0.045 to 0.084
Work-related stress	0.0580	11.3	4	0.042 to 0.071
Illegitimate tasks	0.0468	9.1	5	0.033 to 0.063
Job security	0.0407	7.9	6	0.023 to 0.061
Sense of community	0.0404	7.8	7	0.029 to 0.057
Predictability	0.0395	7.7	8	0.030 to 0.055
Interpersonal conflict	0.0207	4.0	9	0.015 to 0.030
Job control	0.0162	3.1	10	0.009 to 0.028
Overall R^2^	0.515	100.0		

Point estimates from the domin package (version 3.6.0) on the 20 stacked imputed datasets (*N* = 1,321). Standardized dominance is the general dominance statistic as a percentage of the overall R^2^. Higher scores indicate more of the named construct (Table 2). The two leader scales were not administered to senior researchers.

*2.5th to 97.5th percentiles of 500 bootstrap estimates (100 replications in each of imputed datasets 1 to 5).

### 3.3 Dominance analysis of full predictors

The manual calculations for the five highest-ranked predictors reproduced the automated Stata output (Supplementary Table S3–S5). The predictors explained 48.5 and 51.5% of the variance in burnout among junior and senior researchers, respectively (Tables 4, 5; Figure 2). The four most important predictors were identical and ranked in the same order in both groups: resilience, role clarity, work–family conflict, and work-related stress. Resilience was the strongest contributor (25.1% of the explained variance among junior and 23.4% among senior researchers), followed by role clarity (15.0 and 13.6%). Job security ranked fifth among junior researchers (8.0%), whereas illegitimate tasks ranked fifth among senior researchers (9.1%).

**Figure 2:**
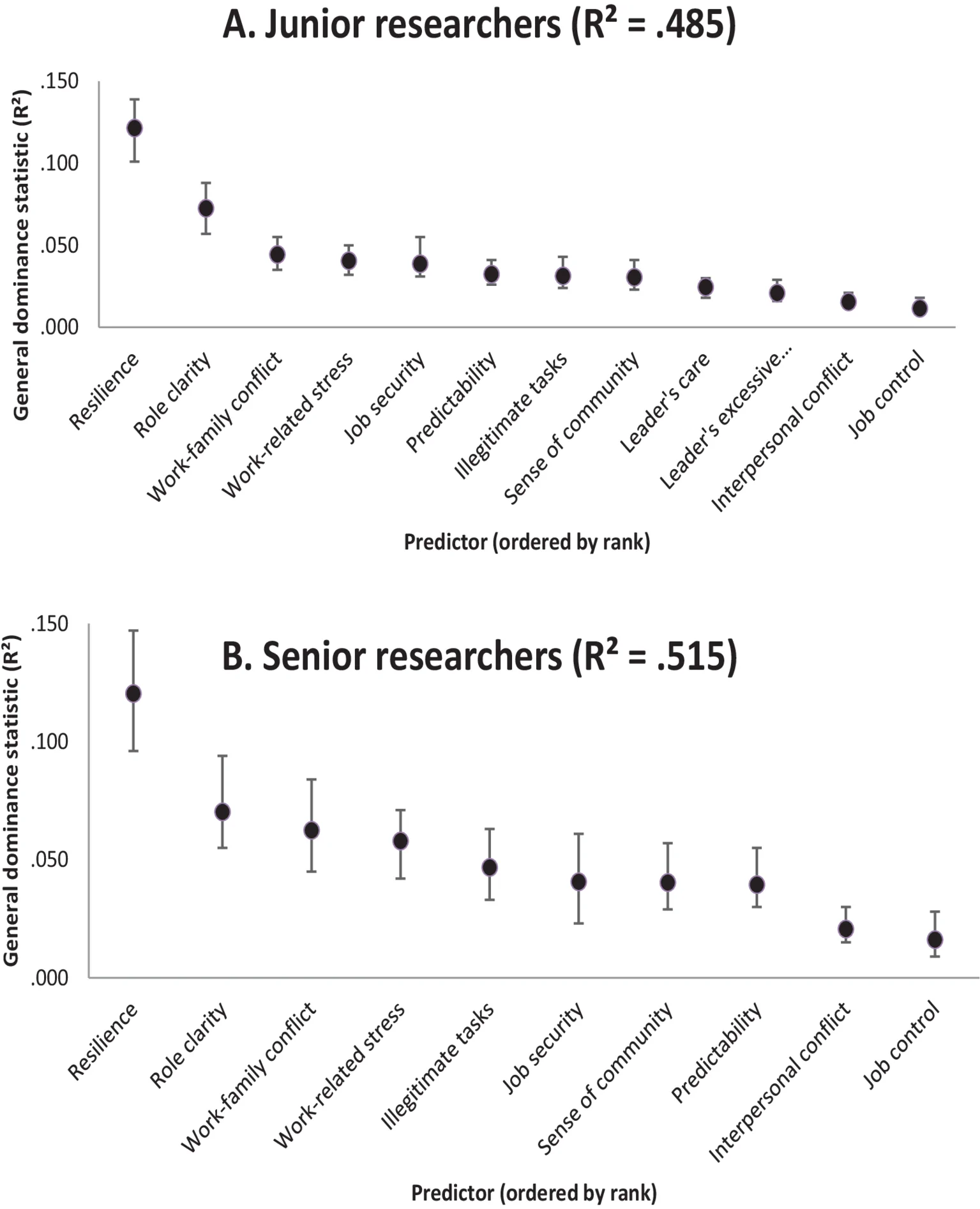
General dominance statistics of burnout predictors among **(A)** junior and **(B)** senior researchers. Dots show the general dominance statistic for each predictor, ordered by rank; vertical lines are 95% bootstrap intervals (2.5th to 97.5th percentiles of 500 bootstrap estimates). R^2^, proportion of variance in burnout explained by all predictors.

All 95% bootstrap intervals excluded zero, but intervals of adjacent predictors frequently overlapped (Figure 2). Among junior researchers, resilience and role clarity were clearly separated from each other and from all other predictors, whereas intervals overlapped among adjacent predictors from rank 3 downward. Among senior researchers, only resilience was clearly separated; the interval for role clarity overlapped with those for work–family conflict and work-related stress, and intervals overlapped among adjacent predictors from rank 2 downward. The exact ordering of predictors below the top two (in junior researchers) or top one (in senior researchers) should therefore not be overinterpreted. When the junior analysis was restricted to the same ten predictors as the senior analysis, the ranking was unchanged: illegitimate tasks ranked seventh among junior researchers (7.6%) compared with fifth among senior researchers (9.1%) (Supplementary Table S6).

Because resilience was the top-ranked predictor and is itself an affectively toned construct, we examined its association with burnout. Resilience correlated −0.47 with overall burnout among junior and −0.50 among senior researchers, and −0.33 to −0.43 with the four burnout dimensions (Supplementary Table S9). A confirmatory factor analysis showed that a two-factor model, with resilience and burnout as separate but correlated factors, fitted considerably better than a one-factor model in the combined sample (Δχ^2^(1) = 3782.67, *p* < 0.001) and among junior (Δχ^2^(1) = 2788.13, *p* < 0.001) and senior (Δχ^2^(1) = 1009.49, *p* < 0.001) researchers, although the absolute fit of the two-factor model remained modest. The latent correlation between the two factors was moderate (combined r = −0.564, 95% CI [−0.592, −0.536]; junior r = −0.548, 95% CI [−0.583, −0.513]; senior r = −0.585, 95% CI [−0.634, −0.537]; Supplementary Table S10), indicating that resilience and burnout are related but empirically distinguishable constructs.

The general dominance ranking was consistent with the conditional dominance results: at every model size, the leading predictors retained their order (Supplementary Tables S11, S12). Complete dominance was more restrictive. Resilience completely dominated all other predictors in both groups, but many pairs, particularly adjacent mid-ranked predictors, did not show complete dominance, consistent with their overlapping bootstrap intervals. Dimension-level analyses (Supplementary Tables S13, S14) showed a broadly similar pattern to the overall-burnout results, with some notable differences by dimension. Resilience ranked first or second across all four BAT-12 dimensions in both junior and senior researchers, confirming that its prominence was not specific to overall burnout. Work–family conflict ranked first for exhaustion in both groups but fell sharply for mental distance (10th among juniors, 8th among seniors), suggesting its relative importance is concentrated in the exhaustion component rather than the more depersonalization-oriented mental distance dimension. Role clarity showed the reverse pattern, ranking first (juniors) or second (seniors) for mental distance while ranking lower for exhaustion (5th and 4th, respectively). Among senior researchers, illegitimate tasks were notably more important for emotional impairment (rank 2) than for overall burnout (rank 5), a pattern not observed among junior researchers.

## 4 Discussion

Using dominance analysis, this study examined the relative importance of psychosocial and organizational predictors of burnout among European researchers at different career stages. Across both junior and senior researchers, resilience and role clarity were the two most prominent predictors, followed by work–family conflict and work-related stress. Consistent with our expectation, demand-related predictors, in particular low role clarity, work–family conflict, and work-related stress, ranked above job control and sense of community; however, resilience, a personal resource, ranked above all job characteristics. Job security ranked slightly higher among junior researchers, whereas illegitimate tasks showed greater relative importance among senior researchers. Importantly, several adjacent mid-ranked predictors had overlapping bootstrap intervals, indicating that their exact ordering should not be overinterpreted. The findings therefore point to a consistent pattern of relative importance rather than a sharply separated hierarchy among all predictors. There is no established threshold for a meaningful difference in dominance statistics, so we used non-overlapping bootstrap intervals as a conservative criterion. By this criterion, the gap between resilience and role clarity is robust: resilience accounted for about 12 percentage points of explained variance and role clarity for about 7, and their intervals did not overlap in either group (juniors 0.101 to 0.139 vs. 0.057 to 0.088; seniors 0.096 to 0.147 vs. 0.055 to 0.094). In practical terms, resilience accounted for roughly 1.7 times as much explained variance as role clarity. By contrast, adjacent mid-ranked predictors differed by less than one percentage point of explained variance, with overlapping intervals, and their order should not be read as a difference in importance. Taken together, the results suggest that burnout among researchers is associated with a combination of individual resources (i.e., resilience) and psychosocial and organizational working conditions, rather than being uniquely accounted for by any single factor.

This finding contributes to a literature that has identified a wide range of psychosocial and organizational correlates of academic burnout but has less often examined their relative importance within a unified analytical framework. A systematic review of university faculty identified multiple determinants, including workload, work–family conflict, job insecurity, and broader organizational and interpersonal factors (8). Similarly, a recent scoping review across healthcare, educational, and other professional populations highlighted the multifactorial nature of burnout and the wide range of psychosocial and organizational factors associated with it (10). Against this background, the present study extends previous research by comparing the relative contribution of multiple correlated predictors simultaneously. This distinction is important because conventional regression coefficients may be difficult to interpret as indicators of relative predictor importance when predictors are correlated. Dominance analysis addresses this issue by evaluating each predictor’s contribution to explained variance across all possible subset models (22). Consistent with this, Gluschkoff et al. found that no single psychosocial factor dominated across all three Maslach Burnout Inventory (MBI) dimensions: time pressure was the strongest predictor of emotional exhaustion, whereas patient-related stress was most important for both depersonalization and reduced personal accomplishment, together accounting for roughly half of the explained variance in each of these two dimensions (34). Our dimension-level results showed a partially different pattern. Resilience ranked first or second across all four BAT-12 dimensions in both junior and senior researchers, indicating a more uniform association than was observed for the dominant predictors in Gluschkoff et al.’s physician sample. Some of our predictors did, however, show the kind of dimension-specific importance that they reported: work–family conflict was the top-ranked predictor for exhaustion in both groups but fell markedly in rank for mental distance, echoing the broader pattern whereby different psychosocial factors dominated different burnout dimensions among physicians. Taken together, these findings highlight that some predictors may operate broadly across burnout dimensions, whereas others are more dimension-specific, supporting the value of examining individual dimensions rather than relying solely on composite burnout scores.

Relative importance studies in other occupational groups point in a different direction. Among 4,591 direct care nurses in European hospitals, Kohnen et al. (35) used the BAT and dominance analysis and found that emotional dissonance (17.0 to 23.8% of explained variance) and emotional demands (17.3 to 20.8%) were the strongest predictors of burnout. That study, however, examined job demands and resources only and did not include personal resources, so it cannot show whether an individual resource would have ranked above them. Among 226 UK teachers, Ainsworth and Oldfield compared eight individual and seven contextual factors using relative importance analysis and found that contextual factors, particularly support from management, school culture, and workload, explained more of the variance in teachers’ burnout, job satisfaction, and well-being than individual factors (36). Taken together, these comparisons suggest that the primacy of an individual resource observed here may not generalize to other occupations. Whether it is specific to academia, or partly reflects the overlap between the Brief Resilience Scale and burnout discussed below, cannot be determined from the present data.

### 4.1 Resilience as the most prominent predictor

The most notable finding was the consistently high relative importance of resilience, which ranked first for overall burnout in both career-stage groups. This pattern was broadly supported by the dimension-level analyses, in which resilience remained among the two highest-ranked predictors across all four BAT dimensions in both groups. Thus, its prominence was not specific to the use of the overall BAT score.

The finding nevertheless requires careful interpretation. The Brief Resilience Scale (BRS) assesses the perceived capacity to recover or “bounce back” from stress, whereas the BAT assesses burnout through exhaustion, mental distance, cognitive impairment, and emotional impairment. Resilience correlated −0.47 with overall burnout among junior and −0.50 among senior researchers (Supplementary Table S9), and the latent correlation between the two constructs was −0.55 to −0.59 (Supplementary Table S10). The two-factor model fitted considerably better than a single-factor model, indicating that resilience and burnout are empirically distinguishable. This does not mean that they overlap little: a latent correlation of this size implies that roughly 30 to 35% of their variance is shared. Because both were self-reported at the same time, and both are likely to carry general negative affect, part of the resilience signal may reflect this shared affective component rather than a resource that is independent of the outcome it predicts. We therefore interpret the high dominance of resilience as its relative contribution to explained variance within the specified predictor set, part of which may reflect variance shared with burnout, rather than as evidence that resilience is a distinct, causally prior resource.

The high dominance of resilience also reflects its position within the predictor set. Dominance statistics capture a predictor’s contribution across subsets of correlated predictors, so a predictor that overlaps little with the others retains more of its explained variance. Resilience correlated only weakly with the other predictors (absolute r = 0.14 to 0.29), whereas several job demands were substantially intercorrelated, for example work-related stress with work–family conflict (r = 0.59 among junior and 0.58 among senior researchers) and with illegitimate tasks (r = 0.50 and 0.57; Supplementary Tables S7, S8). The variance these demands explain is therefore shared among them, whereas resilience claims its contribution largely alone. Similar considerations have motivated the use of dominance analysis in research examining psychosocial predictors of burnout (34).

The prominence of resilience is consistent with research identifying resilience as an important resource in academic populations (14). However, resilience is increasingly conceptualized not simply as a fixed individual trait but as a process shaped by the environments in which people work, involving personal resources, coping, and adaptation to stress (37, 38). This caution is reinforced by the overlap described above: if part of the resilience signal reflects burnout-related affect, a high resilience score may partly indicate the absence of burnout rather than a resource that individuals can build independently of their working conditions. Research specifically examining academics further emphasizes the role of the wider higher-education environment in shaping resilience and adaptation (39). Thus, the present finding should not be interpreted as suggesting that researchers simply need to become more resilient. Rather, resilience may represent an individual resource whose relevance is closely connected to the conditions in which academic work is performed.

### 4.2 Role clarity and the organization of academic work

Role clarity was the second most important predictor in both career-stage groups, and its importance remained relatively high across the BAT dimensions. This finding is particularly relevant to the changing organization of academic work. Contemporary researchers are expected to combine research, teaching, supervision, grant acquisition, administration, institutional service, and increasingly diverse forms of knowledge exchange and communication (40). Such role diversification can create uncertainty about priorities and performance expectations, particularly when different responsibilities are evaluated according to competing criteria.

Previous research supports the importance of these organizational conditions. A systematic review of academic burnout identified workload, organizational conditions, role-related factors, and other psychosocial characteristics as important correlates of burnout among university faculty (8). Earlier research among UK academics similarly documented high job demands and poor work-life balance, while greater job control and institutional support were associated with more favourable working conditions (17). Our findings extend this literature by showing that, among the predictors considered simultaneously, role clarity was consistently more prominent than many other organizational factors. The finding has practical implications. Role ambiguity in academia may reflect not only unclear expectations but also the accumulation of excessive and sometimes conflicting demands across domains, as researchers are often expected to simultaneously excel in research, teaching, supervision, funding acquisition, and institutional service (41). Unlike many other occupations, academic work has no clearly defined upper limit, since there is always more that could be researched, published, or taught. Improving role clarity may therefore involve not only clearer priorities, responsibilities, evaluation criteria, and workload expectations, but also institutions setting explicit boundaries on what is expected, rather than leaving performance standards open-ended. Transparent evaluation systems and clearly defined, bounded responsibilities may help improve role clarity and potentially prevent burnout.

### 4.3 Work-related stress and work–family conflict

Work-related stress and work–family conflict ranked third and fourth in both groups, suggesting that job demands and work-life boundaries are relevant to academic burnout. Their intervals, however, overlapped with those of several other predictors, particularly among junior researchers, so their position relative to the mid-ranked predictors should not be overinterpreted. Work–family conflict has been identified as an important correlate of burnout in studies of university faculty (9) and university employees in Germany (42). These findings are also consistent with broader evidence that burnout reflects multiple psychosocial and organizational conditions (8, 10). Our results extend this literature by showing that work-related stress and work–family conflict remained relatively important when considered simultaneously with resilience, role clarity, job security, illegitimate tasks, and other predictors.

### 4.4 Illegitimate tasks and the changing academic role

Illegitimate tasks showed greater relative importance among senior researchers, ranking fifth and accounting for 9.1% of explained variance, compared with seventh and 6.5% among junior researchers. This difference was not an artefact of the larger junior model: when the analysis of junior researchers was restricted to the same ten predictors as the senior analysis, illegitimate tasks still ranked seventh (7.6%) (Supplementary Table S6). This is consistent with evidence that tasks perceived as unnecessary or unreasonable can be an important source of occupational strain, distinct from workload itself (43). A systematic review further linked illegitimate tasks to a range of health, well-being, cognitive, and work-related outcomes (44). Illegitimate tasks may be particularly relevant in academia, because researchers are increasingly tasked with administrative reporting, compliance, funding administration, and institutional service beyond their core scholarly activities.

Recent qualitative evidence from social science academics provides an academic-specific perspective, showing that illegitimate tasks were associated with role conflict, fatigue, negative emotions, and reduced work commitment, while also highlighting organizational processes and unclear roles as important sources of such tasks (40). Evidence from doctoral and postdoctoral researchers in German universities has also linked illegitimate tasks with burnout facets in the context of ethical leadership (45). These findings provide context for the present results showing a greater relative prominence of illegitimate tasks among senior researchers.

This career-stage difference should nevertheless be interpreted cautiously. Senior researchers may have greater responsibility for supervision, grant and group management, administration, institutional service, and communication which could be associated with greater exposure to tasks perceived as outside their core role. Thus, the observed difference may reflect differences in the nature and organization of academic responsibilities rather than workload alone.

### 4.5 Job security and career stage

Job security ranked fifth among junior researchers and sixth among senior researchers, although its general dominance statistic was marginally larger in absolute terms among seniors (0.039 vs. 0.041; Tables 4, 5). This rank difference reflects illegitimate tasks moving up two positions among seniors (from seventh to fifth), displacing job security, rather than a decline in its own contribution; the senior model also retains only ten predictors compared with twelve for juniors, which mechanically raises each retained predictor’s standardized share of explained variance in that group. The relative prominence of job security among junior researchers is nonetheless consistent with the employment conditions observed in our sample: approximately half of junior researchers held contracts ending within two years, whereas permanent positions were much more common among senior researchers. Lower job security was associated with higher burnout in both groups (Table 3). Precarity has also been identified as an important psychosocial stressor among researchers. Among research fellows in science, technology, engineering, and mathematics (STEM), greater job insecurity was associated with poorer well-being, emotional exhaustion, and cynicism (46).

More broadly, research among PhD students has shown that adverse work and organizational conditions, including high job demands, work–family conflict, limited job control, and concerns about career prospects, are associated with poorer mental health (47). Together, these findings provide context for the relevance of job security to burnout among researchers more broadly, indicating that it makes a meaningful but not exceptional contribution within the predictor set examined for both career stages, rather than being a career-stage-specific concern or the factor whose improvement would necessarily produce the largest reduction in burnout.

### 4.6 What the BAT dimension analyses add

The supplementary analyses of the four BAT dimensions broadly supported the main findings, particularly the consistent prominence of resilience across dimensions in both career-stage groups (Supplementary Tables S13, S14). However, some predictors showed dimension-specific patterns. Among junior researchers, work–family conflict ranked first for exhaustion but tenth for mental distance, while role clarity was more prominent for mental distance, cognitive impairment, and emotional impairment. Among senior researchers, illegitimate tasks ranked second for emotional impairment and job security ranked third for mental distance, compared with fifth and sixth, respectively, for overall burnout. These findings suggest that the overall BAT score provides a useful summary of burnout but also that it may mask differences in the relative importance of predictors across specific burnout manifestations.

This pattern is consistent with dominance-analysis research among physicians, where the relative importance of psychosocial predictors also varied across burnout dimensions (34). These analyses were exploratory and were not intended to formally test differences in predictor importance across BAT dimensions. They nevertheless suggest that some predictors may be more relevant to specific dimensions of burnout than to overall burnout.

### 4.7 Implications for academic organizations and policy

Taken together, the findings suggest that burnout among researchers should not be viewed solely as an individual resilience problem. The prominence of resilience alongside role clarity, work-related stress, work–family conflict, job security, and illegitimate tasks points to the importance of the conditions in which researchers work. This is consistent with a salutogenic perspective, which emphasizes organizational environments that provide resources supporting health and well-being (48, 49).

A Total Worker Health perspective similarly emphasizes addressing well-being through improvements in working conditions and organizational policies (50). Our findings point to concrete measures at three levels, each linked to a predictor that can be monitored. At the department level, heads of department could agree a written annual allocation of research, teaching, supervision, and service time with each researcher, reviewed at the annual appraisal; this targets role clarity. Institutions could audit recurring administrative and reporting tasks with researchers, remove those that are unnecessary, and reassign the remainder to administrative staff; this targets illegitimate tasks, particularly among senior researchers. Institutions could also adopt policies limiting meetings and email expectations outside core working hours and ensure cover for parental and care leave; this targets work–family conflict. At the national level, research funders could set minimum durations for postdoctoral contracts and allow contracts to extend beyond a single grant, for example through bridging funding; this targets job security among junior researchers. To know whether such measures have any effect, institutions could repeat the validated measures used here (role clarity, illegitimate tasks, work–family conflict, job security, and the BAT-12) in staff surveys every one to two years and track changes by career stage. Because our findings are cross-sectional, these measures should be evaluated as interventions rather than assumed to reduce burnout.

Importantly, resilience should be supported within this broader organizational context. Research on academics increasingly conceptualizes resilience as a capacity shaped by the wider higher-education environment and the resources available to individuals (39). Resilience research has also emphasized the role of available resources and coping processes in supporting positive adaptation to academic stress (37). Mentoring, peer support, and resilience-oriented programs may therefore be useful, but they should complement rather than substitute for tangible organizational changes that reduce unnecessary demands and improve predictability, support, and working conditions. Where institutions do offer individual support, it needs to reach researchers. A mapping of occupational health services in 14 Swiss higher education institutions found that psychological counselling was available to students in 13 institutions but to research staff in only nine, and in a pilot study at one institution, researchers were largely unaware of the services available and preferred external help because of concerns about confidentiality (51). Institutions could therefore ensure that confidential psychological support, including externally provided options, is open to research staff at all career stages and actively communicated to them, and could monitor researchers’ awareness and use of these services alongside the working-condition measures described above. Thus, resilience may be considered one component of a healthier academic environment rather than a substitute for structural change.

### 4.8 Strengths and limitations

This study has several strengths. First, the large European sample and stratification by career stage enabled examination of whether the relative importance of burnout predictors was consistent across different stages of academic careers. Second, dominance analysis allowed correlated psychosocial and organizational predictors to be compared on a common metric of relative contribution to explained variance. Third, multiple imputation was used to reduce the loss of information associated with complete-case analysis. Fourth, the robustness of the dominance rankings was examined using bootstrap intervals, a sensitivity analysis with a common predictor set, and supplementary analyses of the four BAT dimensions. Finally, the correlational and confirmatory factor analyses of resilience and burnout clarified the extent of their overlap, which is essential for interpreting the high relative importance of resilience.

Several limitations should nevertheless be considered. First, the cross-sectional design precludes causal inference. Burnout may itself shape perceptions of resilience, role clarity, work stress, and other working conditions, and unmeasured third variables, such as general well-being or negative affectivity, may account for part of the observed associations. Second, all measures were self-reported, which may introduce reporting and common-method bias; this is particularly relevant to resilience, which shared substantial variance with burnout. Third, participation was voluntary, and recruitment took place through open online channels. The number of researchers who were invited or who viewed the survey is therefore unknown, a response rate could not be computed, and the participant flow could be described only from the point at which participants started the survey (Figure 1). Participants may differ systematically from non-participants, and women were overrepresented in both groups, which limits generalizability. Fourth, four initially considered measures were excluded because of high missingness (Supplementary Table S1); three of these, supervisor integrity, emotional support, and instrumental support, are potentially important job resources (18, 19), so the rankings apply to the retained predictors rather than to all possible determinants of burnout. Fifth, measurement invariance across career stages was not established: in a companion analysis of the same data, neither metric nor scalar invariance of a Job Demands-Resources measurement model including the BAT-12 was supported (31), and invariance of the Brief Resilience Scale was not tested. Sixth, participants were clustered in countries with different employment conditions, contract law, and career structures, but this clustering was not modelled, since the country variable was hidden for confidentiality reasons. Clustering affects the precision rather than the point estimates on which the rankings are based, but the bootstrap intervals may be somewhat too narrow. Seventh, although missingness in burnout was not associated with the observed demographic variables, the missing-at-random assumption cannot be verified. Finally, dominance analysis estimates relative statistical rather than causal importance, so the rankings do not indicate that changing the highest-ranked predictor would produce the largest reduction in burnout. The dimension-level analyses were exploratory and did not formally test differences in predictor importance across dimensions.

## 5 Conclusion

This study identified resilience and role clarity as the most prominent predictors of burnout, followed by work–family conflict and work-related stress among European researchers. Resilience remained highly ranked across the four BAT dimensions, but it shared substantial variance with burnout (r = −0.47 to −0.50), and part of its prominence may reflect this overlap. Its dominance should therefore be understood as a relative contribution within the predictor set rather than as evidence of an individual causal effect, and it does not imply that burnout can be addressed by strengthening individual resilience alone.

Career-stage differences were also observed, with illegitimate tasks showing greater relative importance among senior researchers and job security ranking slightly higher among junior researchers. Organizational strategies focusing on clearer roles, sustainable workloads, career stability, and reduction of work stress and illegitimate tasks may therefore represent promising priorities for further evaluation. It should be noted, however, that the relative importance of predictors assessed in the present study was based on cross-sectional data and should not be interpreted as causal effects.

## Data Availability

TThe data analysed in this study are from the ReMO STAIRCASE Survey and are available under restricted access from the Research Data Centre for Higher Education Research and Science Studies (FDZ-DZHW), German Centre for Higher Education Research and Science Studies (DZHW). Anonymised Campus Use Files and pseudonymised Scientific Use Files can be accessed via a secure remote analysis environment: https://doi.org/10.21249/DZHW:remo:1.0.0.
