# Supplementary material for: Relative importance of psychosocial and organizational predictors of burnout among European researchers: a dominance analysis
Source: Front Public Health. 2026 Oct 19;14:1900453. doi: 10.3389/fpubh.2026.1900453 (PMC13724495; doi:10.3389/fpubh.2026.1900453)
Supplement: Supplementary file 1 [file Supplementary_file_1.docx]

**Supplementary File S1**

**STROBE Statement: checklist of items that should be included in reports of cross-sectional studies**

*Relative importance of psychosocial and organizational predictors of burnout among European researchers: a dominance analysis*

| **Section/Topic** | **Item No** | **Recommendation** | **Reported in section** |
| --- | --- | --- | --- |
| **Title and abstract** | 1 | (a) Indicate the study's design with a commonly used term in the title or the abstract | Abstract (Methods) |
|  |  | (b) Provide in the abstract an informative and balanced summary of what was done and what was found | Abstract |
| **Introduction** | | | |
| **Background/rationale** | 2 | Explain the scientific background and rationale for the investigation being reported | 1 Introduction |
| **Objectives** | 3 | State specific objectives, including any prespecified hypotheses | 1 Introduction (final paragraph) |
| **Methods** | | | |
| **Study design** | 4 | Present key elements of study design early in the paper | 2.1 Study population and design |
| **Setting** | 5 | Describe the setting, locations, and relevant dates, including periods of recruitment, exposure, follow-up, and data collection | 2.1 Study population and design; 2.2 Data collection; Figure 1 |
| **Participants** | 6 | (a) Give the eligibility criteria, and the sources and methods of selection of participants | 2.1 Study population and design; 2.2 Data collection; Figure 1 |
| **Variables** | 7 | Clearly define all outcomes, exposures, predictors, potential confounders, and effect modifiers. Give diagnostic criteria, if applicable | 2.3 Study outcome; 2.4 Predictor variables; Table 2 |
| **Data sources/measurement** | 8* | For each variable of interest, give sources of data and details of methods of assessment (measurement). Describe comparability of assessment methods if there is more than one group | 2.3 Study outcome; 2.4 Predictor variables; Table 2 |
| **Bias** | 9 | Describe any efforts to address potential sources of bias | 2.5.2 Missing data handling; 2.5.4 Discriminant validity of resilience and burnout; 4.8 Strengths and limitations |
| **Study size** | 10 | Explain how the study size was arrived at | 2.1 Study population and design; 3.1 Study sample description; Figure 1 |
| **Quantitative variables** | 11 | Explain how quantitative variables were handled in the analyses. If applicable, describe which groupings were chosen and why | 2.3 Study outcome; 2.4 Predictor variables; 2.5.1 Sample stratification and exploratory analyses |
| **Statistical methods** | 12 | (a) Describe all statistical methods, including those used to control for confounding | 2.5.1 Sample stratification and exploratory analyses; 2.5.3 Dominance analysis strategy; 2.5.4 Discriminant validity of resilience and burnout |
|  |  | (b) Describe any methods used to examine subgroups and interactions | 2.4 Predictor variables (separate analyses by career stage); 2.5.3 Dominance analysis strategy (BAT-12 dimensions) |
|  |  | (c) Explain how missing data were addressed | 2.4 Predictor variables; 2.5.2 Missing data handling |
|  |  | (d) If applicable, describe analytical methods taking account of sampling strategy | Not applicable (open online recruitment); see 4.8 Strengths and limitations |
|  |  | (e) Describe any sensitivity analyses | 2.5.1 Sample stratification and exploratory analyses; 2.5.3 Dominance analysis strategy; 2.5.4 Discriminant validity of resilience and burnout |
| **Results** | | | |
| **Participants** | 13* | (a) Report numbers of individuals at each stage of study, e.g., numbers potentially eligible, examined for eligibility, confirmed eligible, included in the study, completing follow-up, and analysed | 3.1 Study sample description; Figure 1 |
|  |  | (b) Give reasons for non-participation at each stage | Figure 1; 3.1 Study sample description |
|  |  | (c) Consider use of a flow diagram | Figure 1 |
| **Descriptive data** | 14* | (a) Give characteristics of study participants (e.g., demographic, clinical, social) and information on exposures and potential confounders | 3.1 Study sample description; Table 1 |
|  |  | (b) Indicate number of participants with missing data for each variable of interest | Table 2; Supplementary Table S1 |
| **Outcome data** | 15* | Report numbers of outcome events or summary measures | 3.1 Study sample description; Table 2; Supplementary Tables S7, S8 |
| **Main results** | 16 | (a) Give unadjusted estimates and, if applicable, confounder-adjusted estimates and their precision (e.g., 95% confidence interval). Make clear which confounders were adjusted for and why they were included | 3.2 Regression results; Table 3; 3.3 Dominance analysis of full predictors; Tables 4, 5; Figure 2 |
|  |  | (b) Report category boundaries when continuous variables were categorized | Not applicable (all variables analysed as continuous) |
|  |  | (c) If relevant, consider translating estimates of relative risk into absolute risk for a meaningful time period | Not applicable |
| **Other analyses** | 17 | Report other analyses done, e.g., analyses of subgroups and interactions, and sensitivity analyses | 3.3 Dominance analysis of full predictors; Supplementary Tables S2 to S6 and S9 to S14 |
| **Discussion** | | | |
| **Key results** | 18 | Summarise key results with reference to study objectives | 4 Discussion (first paragraph) |
| **Limitations** | 19 | Discuss limitations of the study, taking into account sources of potential bias or imprecision. Discuss both direction and magnitude of any potential bias | 4.8 Strengths and limitations |
| **Interpretation** | 20 | Give a cautious overall interpretation of results considering objectives, limitations, multiplicity of analyses, results from similar studies, and other relevant evidence | 4 Discussion; 4.1 to 4.7; 5 Conclusion |
| **Generalisability** | 21 | Discuss the generalisability (external validity) of the study results | 4 Discussion (comparison with other occupational groups); 4.8 Strengths and limitations |
| **Other information** | | | |
| **Funding** | 22 | Give the source of funding and the role of the funders for the present study and, if applicable, for the original study on which the present article is based | Funding |

*Give information separately for exposed and unexposed groups if applicable. In this study, information is given separately for junior and senior researchers.

Section headings refer to the published article. Checklist adapted from: von Elm E, Altman DG, Egger M, Pocock SJ, Gøtzsche PC, Vandenbroucke JP. The Strengthening the Reporting of Observational Studies in Epidemiology (STROBE) statement: guidelines for reporting observational studies. Lancet. 2007;370:1453–7. The STROBE checklist is distributed under the Creative Commons Attribution License.
