# Supplementary material for: Relative importance of psychosocial and organizational predictors of burnout among European researchers: a dominance analysis
Source: Front Public Health. 2026 Oct 19;14:1900453. doi: 10.3389/fpubh.2026.1900453 (PMC13724495; doi:10.3389/fpubh.2026.1900453)
Supplement: Supplementary file 2 [file Table_1.docx]

***Supplementary Table S1. Missing data for the four measures excluded from the analysis because more than 30% of responses were missing***

| **Excluded measure** | **Junior researchers, missing n (%) (N = 2,705)** | **Senior researchers, missing n (%) (N = 1,321)** |
| --- | --- | --- |
| Policy perception | 1,244 (46.0) | 490 (37.1) |
| Emotional support | 1,203 (44.5) | 586 (44.4) |
| Instrumental support | 1,221 (45.1) | 590 (44.7) |
| Supervisor integrity | 967 (35.8) | 987 (74.7) |

*Note.* Missingness at the scale (factor score) level. Even at a 40% threshold, policy perception, emotional support and instrumental support would remain excluded in both groups, as would supervisor integrity among senior researchers; only supervisor integrity among junior researchers (35.8%) would fall below that threshold.

***Supplementary Table S2. Logistic regression diagnostic model predicting burnout missingness (N = 3,942)***

| **Variable** | **Category** | **Log-Odds**  **Coefficient** | **Standard**  **Error** | ***z*** | ***p*-value** | **95% CI** |
| --- | --- | --- | --- | --- | --- | --- |
| Age | 28–33 (vs. <28) | -0.17 | 0.20 | -0.88 | .38 | [-0.56, 0.21] |
|  | 34–39 | 0.10 | 0.23 | 0.43 | .67 | [-0.35, 0.55] |
|  | 40–45 | 0.37 | 0.24 | 1.52 | .13 | [-0.11, 0.85] |
|  | 46–51 | -0.34 | 0.30 | -1.12 | .26 | [-0.93, 0.25] |
|  | 52–57 | -0.39 | 0.35 | -1.11 | .27 | [-1.07, 0.30] |
|  | >57 | 0.18 | 0.32 | 0.57 | .57 | [-0.45, 0.82] |
| Gender | Female (vs. Male) | -0.04 | 0.12 | -0.30 | .76 | [-0.27, 0.20] |
| Position | First stage (PhD candidate) | -0.43 | 0.54 | -0.80 | .42 | [-1.49, 0.63] |
|  | Recognized (Postdoc/Junior) | -0.45 | 0.55 | -0.82 | .41 | [-1.53, 0.63] |
|  | Established (Senior/Assoc. Prof) | -0.32 | 0.55 | -0.57 | .57 | [-1.40, 0.77] |
|  | Leading (Full Professor) | -0.29 | 0.58 | -0.50 | .62 | [-1.43, 0.85] |
| Constant |  | -2.00 | 0.56 | -3.57 | <.01 | [-3.10, -0.90] |

*Note.* The model was evaluated in the unimputed dataset (MI extract 0). The outcome variable was coded as 0 = observed burnout score and 1 = missing burnout score. Reference categories were Male (Gender), <28 years (Age), and junior researchers without a PhD. Overall model fit: likelihood-ratio χ²(11) = 19.18, p = .06; pseudo-R² = .01.

***Supplementary Table S3. Manual dominance analysis of predictors of burnout among junior researchers***

| Subset Model (X) | *R^2^* | *X_1_* | *X_2_* | *X_3_* | *X_4_* | *X_5_* |
| --- | --- | --- | --- | --- | --- | --- |
| Null (*k*=0) |  | .238 | .227 | .185 | .176 | .164 |
| Mean |  | .238 | .227 | .185 | .176 | .164 |
| *K=1* |  |  |  |  |  |  |
| X_1_ | .238 |  | .138 | .088 | .090 | .049 |
| X_2_ | .227 | .149 |  | .117 | .103 | .116 |
| X_3_ | .185 | .141 | .159 |  | .043 | .051 |
| X_4_ | .176 | .152 | .154 | .052 |  | .064 |
| X_5_ | .164 | .123 | .179 | .072 | .076 |  |
| Mean |  | .141 | .158 | .082 | .078 | .070 |
| *K=2* |  |  |  |  |  |  |
| X_1_X_2_ | .376 |  |  | .061 | .057 | .041 |
| X_1_X_3_ | .326 |  | .111 |  | .026 | .011 |
| X_1_X_4_ | .328 |  | .105 | .024 |  | .014 |
| X_1_X_5_ | .287 |  | .130 | .050 | .055 |  |
| X_2_X_3_ | .344 | .093 |  |  | .024 | .042 |
| X_2_X_4_ | .330 | .103 |  | .038 |  | .053 |
| X_2_X_5_ | .343 | .074 |  | .043 | .040 |  |
| X_3_X_4_ | .228 | .124 | .140 |  |  | .036 |
| X_3_X_5_ | .236 | .101 | .150 |  | .028 |  |
| X_4_X_5_ | .240 | .102 | .143 | .024 |  |  |
| Mean |  | .100 | .130 | .040 | .038 | .033 |
| *K=3* |  |  |  |  |  |  |
| X_1_X_2_X_3_ | .437 |  |  |  | .016 | .012 |
| X_1_X_2_X_4_ | .433 |  |  | .020 |  | .016 |
| X_1_X_2_X_5_ | .417 |  |  | .032 | .032 |  |
| X_1_X_3_X_4_ | .352 |  | .101 |  |  | .007 |
| X_1_X_3_X_5_ | .337 |  | .112 |  | .022 |  |
| X_1_X_4_X_5_ | .342 |  | .107 | .017 |  |  |
| X_2_X_3_X_4_ | .368 | .085 |  |  |  | .032 |
| X_2_X_3_X_5_ | .386 | .063 |  |  | .014 |  |
| X_2_X_4_X_5_ | .383 | .066 |  | .017 |  |  |
| X_3_X_4_X_5_ | .264 | .095 | .136 |  |  |  |
| Mean |  | .077 | .114 | .022 | .021 | .017 |
| *k*=4 |  |  |  |  |  |  |
| X_1_X_2_X_3_X_4_ | .453 |  |  |  |  | .008 |
| X_1_X_2_X_3_X_5_ | .449 |  |  |  | .012 |  |
| X_1_X_2_X_4_X_5_ | .449 |  |  | .012 |  |  |
| X_1_X_3_X_4_X_5_ | .359 |  | .102 |  |  |  |
| X_2_X_3_X_4_X_5_ | .400 | .061 |  |  |  |  |
| Mean |  | .061 | .102 | .012 | .012 | .008 |
| Overall  X_1_X_2_X_3_X_4_X_5_ | .461 |  |  |  |  |  |
| Mean | | .123 | .146 | .068 | .065 | .058 |
| % relative dominance | | 26.77% | 31.68% | 14.78% | 14.11% | 12.65% |
| Mean partial dominance | | .095 | .126 | .039 | .037 | .032 |

*Note.* X1 = Role clarity; X2 = Resilience; X3 = Work stress; X4 = Work–family conflict; X5 = Illegitimate tasks

***Supplementary Table S4. Manual dominance analysis of predictors of burnout among senior researchers***

| Subset Model (X) | *R^2^* | *X_1_* | *X_2_* | *X_3_* | *X_4_* | *X_5_* |
| --- | --- | --- | --- | --- | --- | --- |
| Null (*k*=0) |  | .236 | .252 | .197 | .206 | .195 |
| *k*=0 Mean |  | .236 | .252 | .197 | .206 | .195 |
| *k*=1 |  |  |  |  |  |  |
| X_1_ | .236 |  | .148 | .100 | .108 | .073 |
| X_2_ | .252 | .132 |  | .114 | .111 | .121 |
| X_3_ | .197 | .139 | .169 |  | .058 | .053 |
| X_4_ | .206 | .138 | .157 | .049 |  | .072 |
| X_5_ | .195 | .114 | .178 | .055 | .083 |  |
| Mean |  | .131 | .163 | .080 | .090 | .080 |
| *k*=2 |  |  |  |  |  |  |
| X_1_X_2_ | .384 |  |  | .066 | .066 | .054 |
| X_1_X_3_ | .336 |  | .114 |  | .036 | .016 |
| X_1_X_4_ | .344 |  | .106 | .028 |  | .025 |
| X_1_X_5_ | .309 |  | .129 | .043 | .060 |  |
| X_2_X_3_ | .366 | .084 |  |  | .031 | .038 |
| X_2_X_4_ | .363 | .087 |  | .034 |  | .053 |
| X_2_X_5_ | .373 | .065 |  | .031 | .043 |  |
| X_3_X_4_ | .255 | .117 | .142 |  |  | .037 |
| X_3_X_5_ | .250 | .102 | .154 |  | .042 |  |
| X_4_X_5_ | .278 | .091 | .138 | .014 |  |  |
| Mean |  | .091 | .131 | .036 | .046 | .037 |
| *k*=3 |  |  |  |  |  |  |
| X_1_X_2_X_3_ | .450 |  |  |  | .021 | .014 |
| X_1_X_2_X_4_ | .450 |  |  | .021 |  | .022 |
| X_1_X_2_X_5_ | .438 |  |  | .026 | .034 |  |
| X_1_X_3_X_4_ | .372 |  | .099 |  |  | .010 |
| X_1_X_3_X_5_ | .352 |  | .112 |  | .030 |  |
| X_1_X_4_X_5_ | .369 |  | .103 | .013 |  |  |
| X_2_X_3_X_4_ | .397 | .074 |  |  |  | .028 |
| X_2_X_3_X_5_ | .404 | .060 |  |  | .021 |  |
| X_2_X_4_X_5_ | .416 | .056 |  | .009 |  |  |
| X_3_X_4_X_5_ | .292 | .090 | .133 |  |  |  |
| Mean |  | .070 | .112 | .017 | .027 | .019 |
| *k*=4 |  |  |  |  |  |  |
| X_1_X_2_X_3_X_4_ | .471 |  |  |  |  | .009 |
| X_1_X_2_X_3_X_5_ | .464 |  |  |  | .016 |  |
| X_1_X_2_X_4_X_5_ | .472 |  |  | .008 |  |  |
| X_1_X_3_X_4_X_5_ | .382 |  | .098 |  |  |  |
| X_2_X_3_X_4_X_5_ | .425 | .055 |  |  |  |  |
| Mean |  | .055 | .098 | .008 | .016 | .009 |
| Overall  X_1_X_2_X_3_X_4_X_5_ | .480 |  |  |  |  |  |
| Mean | | .117 | .151 | .068 | .077 | .068 |
| % relative dominance | | 24.28% | 31.47% | 14.07% | 16.03% | 14.14% |
| Mean partial dominance | | .087 | .126 | .035 | .045 | .036 |

*Note.* X1 = Role clarity; X2 = Resilience; X3 = Work stress; X4 = Work–family conflict; X5 = Illegitimate tasks

***Supplementary Table S5. Automated dominance analysis (domin) of the five highest-ranked predictors of burnout among junior and senior researchers***

| Predictor | Junior researchers | | | Senior researchers | | |
| --- | --- | --- | --- | --- | --- | --- |
|  | Domin (R²) | % of R² | Rank | Domin (R²) | % of R² | Rank |
| Resilience | .146 | 31.61% | 1 | .151 | 31.54% | 1 |
| Role clarity | .123 | 26.79% | 2 | .117 | 24.32% | 2 |
| Work stress | .068 | 14.82% | 3 | .068 | 14.06% | 5 |
| Work–family conflict | .065 | 14.15% | 4 | .077 | 15.98% | 3 |
| Illegitimate tasks | .058 | 12.64% | 5 | .068 | 14.09% | 4 |

*Note:* Domin (R²) represents the average marginal contribution of each predictor. % of R² reflects the proportion of total explained variance attributable to each predictor. Dominance statistics were computed using the domin package (version 3.6.0) in Stata and were compared with the manual calculations in Supplementary Tables S3 and S4.

***Supplementary Table S6. General dominance among junior researchers restricted to the ten predictors assessed in both groups, compared with senior researchers***

| **Predictor** | **Junior (10 predictors), GD** | **Share** | **Rank** | **Senior, GD** | **Share** | **Rank** |
| --- | --- | --- | --- | --- | --- | --- |
| Resilience | .1238 | 25.6% | 1 | .1204 | 23.4% | 1 |
| Role clarity | .0799 | 16.5% | 2 | .0703 | 13.6% | 2 |
| Work-family conflict | .0488 | 10.1% | 3 | .0625 | 12.1% | 3 |
| Work-related stress | .0462 | 9.6% | 4 | .0580 | 11.3% | 4 |
| Job security | .0400 | 8.3% | 5 | .0407 | 7.9% | 6 |
| Predictability | .0390 | 8.1% | 6 | .0395 | 7.7% | 8 |
| Illegitimate tasks | .0368 | 7.6% | 7 | .0468 | 9.1% | 5 |
| Sense of community | .0354 | 7.3% | 8 | .0404 | 7.8% | 7 |
| Interpersonal conflict | .0191 | 4.0% | 9 | .0207 | 4.0% | 9 |
| Job control | .0143 | 3.0% | 10 | .0162 | 3.2% | 10 |
| Overall R² | .4834 | 100% |  | .5155 | 100% |  |

*Note*. GD, general dominance statistic; Share, standardized dominance (percentage of the model R²). The junior model excludes leader’s excessive pressure for results and leader’s care, which were not administered to senior researchers, so that both groups have the same ten predictors. Point estimates from the 20 stacked imputed datasets. Illegitimate tasks ranked 7th among junior and 5th among senior researchers with the same predictor set.

***Supplementary Table S7. Correlations among burnout and the twelve predictors, junior researchers***

| **Variable** | **1** | **2** | **3** | **4** | **5** | **6** | **7** | **8** | **9** | **10** | **11** | **12** |
| --- | --- | --- | --- | --- | --- | --- | --- | --- | --- | --- | --- | --- |
| 1. Burnout |  |  |  |  |  |  |  |  |  |  |  |  |
| 2. Resilience | −.475 |  |  |  |  |  |  |  |  |  |  |  |
| 3. Leader’s excessive pressure for results | .370 | −.151 |  |  |  |  |  |  |  |  |  |  |
| 4. Leader’s care | −.383 | .165 | −.634 |  |  |  |  |  |  |  |  |  |
| 5. Work-related stress | .421 | −.196 | .534 | −.317 |  |  |  |  |  |  |  |  |
| 6. Predictability | −.423 | .178 | −.465 | .567 | −.365 |  |  |  |  |  |  |  |
| 7. Role clarity | −.487 | .233 | −.359 | .468 | −.296 | .585 |  |  |  |  |  |  |
| 8. Job security | −.350 | .215 | −.162 | .188 | −.265 | .260 | .254 |  |  |  |  |  |
| 9. Job control | −.269 | .146 | −.360 | .359 | −.274 | .316 | .287 | .165 |  |  |  |  |
| 10. Interpersonal conflict | .334 | −.138 | .469 | −.497 | .347 | −.491 | −.346 | −.226 | −.295 |  |  |  |
| 11. Work-family conflict | .415 | −.223 | .432 | −.269 | .592 | −.309 | −.265 | −.247 | −.183 | .332 |  |  |
| 12. Sense of community | −.376 | .206 | −.322 | .475 | −.220 | .454 | .412 | .202 | .257 | −.516 | −.217 |  |
| 13. Illegitimate tasks | .401 | −.146 | .500 | −.421 | .496 | −.499 | −.413 | −.156 | −.285 | .446 | .429 | −.335 |

*Note.* Pearson correlations on the 20 stacked imputed datasets (N = 54,100 rows; 2,705 respondents). p-values and confidence intervals are not reported because they are not valid on stacked data. Higher scores indicate more of the named construct; job security and leader’s care are keyed so that higher scores indicate greater security and greater care.

***Supplementary Table S8. Correlations among burnout and the ten predictors, senior researchers***

| **Variable** | **1** | **2** | **3** | **4** | **5** | **6** | **7** | **8** | **9** | **10** |
| --- | --- | --- | --- | --- | --- | --- | --- | --- | --- | --- |
| 1. Burnout |  |  |  |  |  |  |  |  |  |  |
| 2. Resilience | −.502 |  |  |  |  |  |  |  |  |  |
| 3. Work-related stress | .445 | −.228 |  |  |  |  |  |  |  |  |
| 4. Predictability | −.428 | .210 | −.328 |  |  |  |  |  |  |  |
| 5. Role clarity | −.485 | .267 | −.284 | .572 |  |  |  |  |  |  |
| 6. Job security | −.339 | .212 | −.105 | .224 | .280 |  |  |  |  |  |
| 7. Job control | −.283 | .214 | −.160 | .261 | .325 | .272 |  |  |  |  |
| 8. Interpersonal conflict | .344 | −.244 | .278 | −.455 | −.349 | −.180 | −.172 |  |  |  |
| 9. Work-family conflict | .454 | −.255 | .580 | −.318 | −.283 | −.155 | −.138 | .292 |  |  |
| 10. Sense of community | −.404 | .293 | −.180 | .474 | .412 | .232 | .295 | −.554 | −.220 |  |
| 11. Illegitimate tasks | .443 | −.201 | .567 | −.463 | −.400 | −.147 | −.267 | .410 | .445 | −.306 |

*Note.* Pearson correlations on the 20 stacked imputed datasets (N = 26,420 rows; 1,321 respondents). p-values and confidence intervals are not reported because they are not valid on stacked data. Higher scores indicate more of the named construct; job security is keyed so that higher scores indicate greater security.

***Supplementary Table S9. Pearson correlations between resilience and overall burnout and BAT-12 dimensions, by career stage***

| **Outcome** | **Junior researchers, r** | **Senior researchers, r** |
| --- | --- | --- |
| Overall burnout (BAT-12) | −.47 | −.50 |
| Exhaustion | −.40 | −.43 |
| Mental distance | −.33 | −.42 |
| Cognitive impairment | −.33 | −.34 |
| Emotional impairment | −.38 | −.37 |
| BRS-6 Cronbach’s alpha | .85 | .81 |

*Note.* Pairwise Pearson correlations on unimputed data (junior n = 2,484 to 2,493; senior n = 1,195 to 1,199); all p < .001. Resilience is the mean of the six Brief Resilience Scale items (BRS-6). On the stacked imputed data, the correlation with overall burnout was −.475 among junior and −.502 among senior researchers. These values replace those reported in the previous version of this table, which were mislabelled regression coefficients.

***Supplementary Table S10. One-factor versus two-factor confirmatory factor analysis of resilience and burnout, overall sample and by career stage***

| Sample | Model | χ² (df) | RMSEA | CFI | TLI | SRMR | AIC | BIC |
| --- | --- | --- | --- | --- | --- | --- | --- | --- |
| Combined | One-factor | 12492.4 (135) | .161 | .578 | .522 | .109 | 162079.5 | 162412.7 |
| Combined | Two-factor | 8709.7 (134) | .135 | .707 | .666 | .079 | 158298.8 | 158638.2 |
| Junior | One-factor | 8873.0 (135) | .165 | .558 | .499 | .114 | 109449.4 | 109761.0 |
| Junior | Two-factor | 6084.9 (134) | .137 | .699 | .656 | .082 | 106663.3 | 106980.7 |
| Senior | One-factor | 3514.4 (135) | .149 | .629 | .580 | .100 | 50574.7 | 50846.5 |
| Senior | Two-factor | 2504.9 (134) | .125 | .740 | .703 | .072 | 49567.2 | 49844.0 |

*Note*. Δχ² tests (Δdf = 1) comparing one- vs. two-factor models: combined Δχ² = 3782.67; junior Δχ² = 2788.13; senior Δχ² = 1009.49; all p < .001. Latent correlations between Resilience and Burnout in the two-factor model: combined r = −.564, 95% CI [−.592, −.536]; junior r = −.548, 95% CI [−.583, −.513]; senior r = −.585, 95% CI [−.634, −.537].

***Supplementary Table S11. Conditional and complete dominance analysis of predictors of burnout among junior researchers***

| Predictors | **Conditional dominance analysis: number of predictors in model (k)** | | | | | | | | | | | |
| --- | --- | --- | --- | --- | --- | --- | --- | --- | --- | --- | --- | --- |
|  | 1 | 2 | 3 | 4 | 5 | 6 | 7 | 8 | 9 | 10 | 11 | 12 |
| Role clarity (X_1_) | .240 | .140 | .100 | .070 | .060 | .050 | .040 | .040 | .030 | .030 | .030 | .020 |
| Resilience (X_2_) | .230 | .170 | .140 | .130 | .120 | .110 | .100 | .100 | .090 | .090 | .090 | .080 |
| Predictability (X_3_) | .180 | .090 | .050 | .030 | .020 | .010 | .010 | <.010 | <.010 | <.010 | <.010 | <.010 |
| Illegitimate tasks (X_4_) | .160 | .080 | .040 | .030 | .020 | .010 | .010 | .010 | .010 | <.010 | <.010 | <.010 |
| Work-related stress (X_5_) | .190 | .100 | .060 | .040 | .030 | .030 | .020 | .020 | .010 | .010 | .010 | .010 |
| Work-family conflict (X_6_) | .180 | .100 | .060 | .050 | .040 | .030 | .020 | .020 | .020 | .010 | .010 | .010 |
| Job security (X_7_) | .120 | .070 | .050 | .040 | .030 | .020 | .020 | .020 | .020 | .010 | .010 | .010 |
| Job control (X_8_) | .070 | .030 | .010 | .010 | <.010 | <.010 | <.010 | <.010 | <.010 | <.010 | <.010 | <.010 |
| Interpersonal conflict (X_9_) | .120 | .040 | .020 | .010 | <.010 | <.010 | <.010 | <.010 | <.010 | <.010 | <.010 | <.010 |
| Sense of community (X_10_) | .140 | .070 | .040 | .030 | .020 | .020 | .010 | .010 | .010 | .010 | .010 | <.010 |
| Leader’s care (X_11_) | .150 | .070 | .040 | .020 | .010 | .010 | .010 | <.010 | <.010 | <.010 | <.010 | <.010 |
| Leader’s excessive pressure for results (X_12_) | .140 | .060 | .030 | .010 | .010 | <.010 | <.010 | <.010 | <.010 | <.010 | <.010 | <.010 |
| Complete dominance analysis | | | | | | | | | | | | |
| Predictors | X_1_ | X_2_ | X_3_ | X_4_ | X_5_ | X_6_ | X_7_ | X_8_ | X_9_ | X_10_ | X_11_ | X_12_ |
| Role clarity (X_1_) | — | .000 | 1.00 | 1.00 | .950 | .920 | 1.00 | 1.00 | 1.00 | 1.00 | 1.00 | 1.00 |
| Resilience (X_2_) | 1.00 | — | 1.00 | 1.00 | 1.00 | 1.00 | 1.00 | 1.00 | 1.00 | 1.00 | 1.00 | 1.00 |
| Predictability (X_3_) | .000 | .000 | — | .310 | .200 | .120 | .120 | .840 | .880 | .200 | .590 | .760 |
| Illegitimate tasks (X_4_) | .000 | .000 | .690 | — | .120 | .070 | .080 | 1.00 | 1.00 | .360 | .780 | 1.00 |
| Work-related stress (X_5_) | .050 | .000 | .800 | .880 | — | .130 | .460 | 1.00 | 1.00 | .710 | .910 | 1.00 |
| Work-family conflict (X_6_) | .080 | .000 | .880 | .930 | .870 | — | .580 | 1.00 | 1.00 | .830 | .930 | 1.00 |
| Job security (X_7_) | .000 | .000 | .880 | .920 | .540 | .420 | — | 1.00 | 1.00 | .900 | .950 | .990 |
| Job control (X_8_) | .000 | .000 | .160 | .000 | .000 | .000 | .000 | — | .650 | .000 | .100 | .490 |
| Interpersonal conflict (X_9_) | .000 | .000 | .120 | .000 | .000 | .000 | .000 | .350 | — | .010 | .060 | .340 |
| Sense of community (X_10_) | .000 | .000 | .800 | .640 | .290 | .170 | .100 | 1.00 | .990 | — | .930 | .890 |
| Leader’s care (X_11_) | .000 | .000 | .410 | .220 | .090 | .070 | .050 | .900 | .940 | .070 | — | .770 |
| Leader's excessive pressure for results (X_12_) | .000 | .000 | .240 | .000 | .000 | .000 | .010 | .510 | .660 | .110 | .230 | — |

*Note.* Conditional dominance: columns give the number of predictors in the model (k); values are the average increase in R² contributed by the row predictor across all models of that size. Complete dominance: values indicate the proportion in which the row predictor contributed more R² than the column predictor across all subset models in which both could be compared (1.00 = always; .000 = never). Job security and leader's care are keyed so that higher scores indicate greater security and greater care.

***Supplementary Table S12. Conditional and complete dominance analysis of predictors of burnout among senior researchers***

| **Conditional dominance analysis: number of predictors in model (k)** | | | | | | | | | | |
| --- | --- | --- | --- | --- | --- | --- | --- | --- | --- | --- |
| Predictor | 1 | 2 | 3 | 4 | 5 | 6 | 7 | 8 | 9 | 10 |
| Role clarity (X_1_) | .240 | .140 | .090 | .060 | .050 | .040 | .030 | .020 | .020 | .020 |
| Resilience (X_2_) | .250 | .170 | .140 | .120 | .100 | .090 | .090 | .080 | .080 | .070 |
| Predictability (X_3_) | .190 | .090 | .050 | .030 | .020 | .010 | .010 | <.010 | <.010 | <.010 |
| Illegitimate tasks (X_4_) | .190 | .100 | .060 | .040 | .020 | .020 | .010 | .010 | .010 | <.010 |
| Work-related stress (X_5_) | .200 | .110 | .080 | .050 | .040 | .030 | .020 | .020 | .010 | .010 |
| Work-family conflict (X_6_) | .210 | .120 | .080 | .060 | .040 | .030 | .030 | .020 | .020 | .010 |
| Job security (X_7_) | .120 | .070 | .050 | .040 | .030 | .030 | .020 | .020 | .020 | .020 |
| Job control (X_8_) | .080 | .040 | .020 | .010 | .010 | <.010 | <.010 | <.010 | <.010 | <.010 |
| Interpersonal conflict (X_9_) | .120 | .050 | .020 | .010 | <.010 | <.010 | <.010 | <.010 | <.010 | <.010 |
| Sense of community (X_10_) | .160 | .080 | .050 | .030 | .020 | .020 | .010 | .010 | .010 | .010 |
| **Complete dominance analysis** | | | | | | | | | | |
| Predictor | X_1_ | X_2_ | X_3_ | X_4_ | X_5_ | X_6_ | X_7_ | X_8_ | X_9_ | X_10_ |
| Role clarity (X_1_) | — | .000 | 1.00 | .860 | .640 | .610 | .910 | 1.00 | 1.00 | 1.00 |
| Resilience (X_2_) | 1.00 | — | 1.00 | 1.00 | 1.00 | 1.00 | 1.00 | 1.00 | 1.00 | 1.00 |
| Predictability (X_3_) | .000 | .000 | — | .350 | .150 | .140 | .130 | .890 | .960 | .300 |
| Illegitimate tasks (X_4_) | .140 | .000 | .650 | — | .090 | .030 | .280 | .890 | .950 | .410 |
| Work-related stress (X_5_) | .360 | .000 | .850 | .910 | — | .360 | .520 | .970 | .990 | .730 |
| Work-family conflict (X_6_) | .390 | .000 | .860 | .970 | .640 | — | .500 | 1.00 | 1.00 | .800 |
| Job security (X_7_) | .090 | .000 | .870 | .720 | .480 | .500 | — | 1.00 | .990 | .890 |
| Job control (X_8_) | .000 | .000 | .110 | .110 | .030 | .000 | .000 | — | .720 | .000 |
| Interpersonal conflict (X_9_) | .000 | .000 | .040 | .050 | .010 | .000 | .010 | .280 | — | .000 |
| Sense of community (X_10_) | .000 | .000 | .700 | .590 | .270 | .200 | .110 | 1.00 | 1.00 | — |

*Note.* Conditional dominance: columns give the number of predictors in the model (k); values are the average increase in R² contributed by the row predictor across all models of that size. Complete dominance: values indicate the proportion in which the row predictor contributed more R² than the column predictor across all subset models in which both could be compared (1.00 = always; .000 = never). Job security is keyed so that higher scores indicate greater security.

***Supplementary Table S13. Dominance analysis across overall burnout and the four BAT dimensions among junior researchers***

| Predictor | Overall Burnout | Exhaustion | Mental Distance | Cognitive Impairment | Emotional Impairment |
| --- | --- | --- | --- | --- | --- |
| Resilience | 1 | 2 | 2 | 1 | 1 |
| Role clarity | 2 | 5 | 1 | 2 | 2 |
| Work-family conflict | 3 | 1 | 10 | 5 | 4 |
| Work-related stress | 4 | 3 | 8 | 4 | 3 |
| Job security | 5 | 8 | 5 | 3 | 8 |
| Predictability | 6 | 6 | 4 | 7 | 9 |
| Illegitimate tasks | 7 | 4 | 6 | 9 | 6 |
| Sense of community | 8 | 10 | 7 | 6 | 5 |
| Leader’s care | 9 | 9 | 3 | 8 | 11 |
| Leader's excessive pressure for results | 10 | 7 | 9 | 10 | 10 |
| Interpersonal conflict | 11 | 11 | 12 | 11 | 7 |
| Job control | 12 | 12 | 11 | 12 | 12 |

*Note.* Values are ranks (1 = highest) of the general dominance statistics for overall burnout and for each BAT-12 dimension, with the same twelve predictors. Job security and leader’s care are keyed so that higher scores indicate greater security and greater care*.*

***Supplementary Table S14. Dominance analysis across overall burnout and the four BAT dimensions among senior researchers***

| Predictor | Overall Burnout | Exhaustion | Mental Distance | Cognitive Impairment | Emotional Impairment |
| --- | --- | --- | --- | --- | --- |
| Resilience | 1 | 2 | 1 | 1 | 1 |
| Role clarity | 2 | 4 | 2 | 2 | 5 |
| Work-family conflict | 3 | 1 | 8 | 4 | 6 |
| Work-related stress | 4 | 3 | 7 | 3 | 4 |
| Illegitimate tasks | 5 | 5 | 6 | 6 | 2 |
| Job security | 6 | 7 | 3 | 5 | 10 |
| Sense of community | 7 | 8 | 4 | 8 | 3 |
| Predictability | 8 | 6 | 5 | 7 | 8 |
| Interpersonal conflict | 9 | 9 | 10 | 9 | 7 |
| Job control | 10 | 10 | 9 | 10 | 9 |

*Note.* Values are ranks (1 = highest) of the general dominance statistics for overall burnout and for each BAT-12 dimension, with the same ten predictors. Job security is keyed so that higher scores indicate greater security.
